# Cases in Private Practice

**Published:** 1871-01

**Authors:** Jno. E. Owens

**Affiliations:** Chicago


Article VII.— *Cases in Private Practice.* By Jno. E. Owens, M.D., Chicago.

*Foreign Body in the Appendix Cæci, with Inflammation, Perforation, and Death.*

Saturday, Dec. 8, 1866. A call was received to visit M. X., aet. 20, at his place of business. The message was not received for some hours. In the meantime another physician was called, who prescribed some medicine for internal use, and mustard externally. In consequence of this, I did not examine the case, but imagined it to be one of ordinary colic.

Sunday, Dec. 9. I was requested to visit the patient at his residence. He was in bed and suffering intense pain in the abdomen to the right of the median line. The patient was well nourished and had been in the enjoyment of health to the date of his present illness. On Saturday, 8th, he and one of his comrades were wrestling, but it seems that the patient did not exert himself very much. On Friday, 7th, he ate some chestnuts—not more than seven or eight. The same evening, he and some friends went out to walk, but the former soon returned, in consequence of pain in his bowels, which moved on Friday, 7th. That which passed, was scanty and hard, and its evacuation required considerable voluntary tenesmus.

The patient (Sunday, Dec. 9th) was found in the following condition: There was an irregular tumor in the right iliac region, extending about one inch to the left of the median line; its upper limit reaching a transverse line drawn two inches below the umbilicus; its breadth being about three and a half inches. The tumor, raised somewhat above the surrounding surface, offered considerable resistance to pressure, was exquisitely tender and painful, and dull on percussion, the surroundings being somewhat tympanitic. The tongue was thickly coated, skin dry, pulse rather full and beating from 115 to 120; the bowels had not moved since Friday, 7th. There was nausea with some vomiting on Saturday, 8th. Ol. Tiglii was prescribed, of which he took about eight drops in two hours. Having remained with him to note the result of the oil, and having found that the bowels did not respond, I gave two large injections of soapsuds, castor-oil with a little salt. These came away in due time, bringing with them a few small, firm, yellowish pieces of fecal matter, containing pieces of chestnuts. When the injections were passing away, he cried, "O, if I could only strain!" I diagnosed intussusception. During this treatment the patient vomited once, but the ejecta were not of a stercoraceous character. Fomentations were applied over the bowels. This afternoon., at 4 o'clock, a consultation was held. In the meantime, there had been a greenish watery evacuation, containing mucus, and attended with considerable pain. The abdomen had grown more tympanitic. At the consultation there was an agreement as to the probable existence of intussusception of the bowel, but the diagnosis was not so positive as to justify any operative interference. The negative symptoms were, the alvine discharge, that had occurred since the last visit; the absence of stercoraceous vomiting; and the still more masked character of the case by the extending peritonitis.

Dec. 9th, 9 o'clock, P. M. He has had, from the bowels, four discharges, of the same character as the last one. The fecal smell was slight, and the operations were attended with less pain, the patient being somewhat under the influence of opium.

Dec. 10th, 8 o'clock, A. M. Patient bathed in a profuse perspiration; hands cold; feet warm; circulation, upon the surface of the body, sluggish; pulse small and feeble, beating 125; vomited some last night. The ejecta from both stomach and bowels were greenish and watery, containing considerable mucus. The substance vomited had no fecal smell; that passed per rectum had very little.

Dec. 10th, 12 o'clock, M. Tenderness more extended, suppression of urine since yesterday.

Dec. 11th. Abdomen more distended; patient passed water; urination being attended with great pain. Since yesterday, three unsuccessful attempts have been made to apply leeches; directed that a blister 4×4 be applied to the abdomen.

Dec. 12th, A. M. Distention increased; tongue moist; pulse 114, of moderate strength; bowels moved last night; character of stool same as the previous ones, but with less mucus; vomited once this morning.

Dec. 12th, 4 P. M. Vomited about a pint of greenish fluid without fecal smell; tongue dry, abdomen very tympanitic, pulse 118.

Dec. 13th. Abdomen very tense, and exquisitely tender.

Dec. 14th. Since last date vomiting has grown more frequent; the eyes more and more sunken; the abdomen more tense; the patient more restless, tearing the flesh here and there with the finger nails; the tongue dryer. Death occurred to-day at 2 o'clock P. M.

Autopsy, seven and a half hours after death. Extensive peritonitis about the cæcum, the lower part of ascending colon, the lower coils of small intestines, accompanied with considerable agglutination. The adhesion of one or two coils of intestines to the urinary bladder accounts for the intense pain felt upon voiding this organ (bladder.) The contracting bladder produced a traction upon the inflamed peritoneum. The mucous coat in some parts of the intestines was inflamed and softened. The cavity of the abdomen contained about one and a half pints of chalky fluid. The vermiform appendix, containing pieces of chestnut, was a mass of inflammation, and was perforated in several places, so that the fluid contents of the bowels could readily escape into the abdominal cavity; a portion of the mesentery was likewise inflamed and softened.

*Cæcitis with Partial Peritonitis. Recovery.*

J. H., a stout and square-built Dane, æt. 21, having light hair, blue eyes, florid complexion, came under my care, Nov. 10th, 1870. Two days before, he experienced chilly sensations followed by pain in the right iliac region. The bowels had not been deranged. He remained at work the day of the attack, but in the evening, immediately after commencing to eat his supper, the pain suddenly became excruciating, and remained so for the greater part of the night. It was accompanied with nausea and vomiting.

Nov. 9th. Patient vomited considerably; pain less severe than that of the night before.

Nov. 10th. The date of the first visit, there was great tenderness in the right iliac region. A mixture of chloroform, laudanum and mucilage was ordered for internal use, and locally hop poultices. This evening the patient took three or four tablespoonsful of gruel, which caused considerable distress through the bowels. This was the first food taken since the evening of the 8th.

Nov. 11th. The bowels having been constipated since the 7th inst. moved to day; tongue somewhat furred, but moist; the face cool; pulse 103, and full; respiration easy and regular; slept but little last night; ordered Chloral Hydrate grs. xxx as a substitute for the first mixture. Hop poultices continued.

Nov. 12th. Took the first dose of Chloral Hydrate at 9 o'clock last evening, and went to sleep in a few moments and slept till 4 o'clock this morning. Pulse 84; respiration 18; tongue cleaner; skin cool; the part less tender and somewhat dull upon percussion, but there is still a faint resonance. The painful part, is a circumscribed swelling in the right iliac region. A line drawn from the middle of Poupart's ligament to the umbilicus, passes close to the center of the swelling. There was a scanty movement of the bowels this morning. The discharge was dark, but contained neither mucus nor blood, and its passage was unattended with pain. The patient has eaten a dish of soup to-day without any resulting distress. In four or five days more, the patient was very comfortable; a little soreness in the right iliac region still remained.

(To be continued.)

---

### *Sulphite of Soda in Erysipelas.*

Dr. Hewson uses this salt applied on lint in a solution of ten grains to the ounce of water, and has obtained most satisfactory results. He supposes that erysipelas is due to cryptogamic or animalcular origin, and the well-known power of the sulphites in destroying such organisms accounts for its useful action in this disease. It invariably destroys all traces of the affection in forty-eight hours after the first application.—*Drug Circular.*

